# Nitrogen Promotes the Salt-Gathering Capacity of *Suaeda salsa* and Alleviates Nutrient Competition in the Intercropping of *Suaeda salsa*/*Zea mays* L.

**DOI:** 10.3390/ijms232415495

**Published:** 2022-12-07

**Authors:** Shoule Wang, Shaoqing Ge, Wenxuan Mai, Changyan Tian

**Affiliations:** 1 State Key Laboratory of Desert and Oasis Ecology, Xinjiang Institute of Ecology and Geography, Chinese Academy of Sciences, Urumqi 830011, China; 2 Shandong Institute of Pomology, Shandong Academy of Agricultural Sciences, Taian 271000, China; 3 University of Chinese Academy of Sciences, Beijing 100049, China

**Keywords:** intercropping, nitrogen forms, *Suaeda salsa*, saline soil

## Abstract

Nitrogen accelerates salt accumulation in the root zone of an euhalophyte, which might be beneficial for inhibiting the salt damage and interspecific competition for nutrients of non-halophytes in intercropping. However, the variations in the effect of euhalophyte/non-halophyte intercropping with nitrogen supply are poorly understood. Here, we selected the euhalophyte *Suaeda salsa* (suaeda) and non-halophyte *Zea mays* L. (maize) as the research objects, setting up three cropping patterns in order to explore the influence of nitrogen application on the intercropping effect in the suaeda/maize intercropping. The results showed that the biomass of maize in the intercropping was significantly lower than that in the monoculture, while for suaeda, it was higher in the intercropping than that in the monoculture. The biomass of maize under NO_3_^−^-N treatment performed significantly higher than that under no nitrogen treatment. Moreover, under suitable NO_3_^−^-N treatment, more salt ions (Na^+^, K^+^) gathered around the roots of suaeda, which weakened the salt damage on maize growth. In the intercropping, the effect of NO_3_^−^-N on the maize growth was enhanced when compared with the non-significant effect of NH_4_^+^-N, but a positive effect of NH_4_^+^-N on suaeda growth was found. Therefore, the disadvantage of maize growth in the intercropping suaeda/maize might be caused by interspecific competition to a certain extent, providing an effective means for the improvement of saline–alkali land by phytoremediation.

## 1. Introduction

Euhalophytes can remove more Na^+^ and Cl^−^ in high salinity environments by a special structure (succulence and compartment) and gene expression so as to resist ionic toxicity in leaves [1,2,3,4,5]. Wang et al. (2020) found that one-year salt extraction by *S. salsa* was approximately 162 kg ha^−1^, especially in the topsoil layer, suggesting that consecutive cultivation of euhalophytes is an efficient method for reclaiming saline soil [6]. Its roots possess the ability to gather more salt ions by rhizosphere activities, which is a vital indicator to evaluate salt-tolerance [7,8,9,10]. Generally, the optimal salt level for euhalophytes is 200–400 mM, which is severely unfavorable for crop root development [11,12]. The physiological changes of glycophytes in the response to salinity stress were clearly reflected due to the root–soil interaction [13]. Developing an effective strategy to elevate the salt-gathering ability of euhalophytes and reduce the damage of high salinity on non-halophytes is a significant factor in the process of realizing the efficient improvement of saline–alkali land.

Intercropping has been extensively adopted worldwide, being conducive to optimizing plantation structure and land use patterns [14,15]. This also brings crops nutrient-competitive advantages and superior yield by interspecific interactions. Rhizosphere dialogues, e.g., ions shift, signal delivery, and material transformation, are certainly present in this complex system [16,17,18]. Under high salinity, halophytes and non-halophytes might separately generate two distinct, different rhizosphere effects: non-halophyte root exudates reduce the soil pH to increase the soil nutrient availability, while halophytes improve salt-tolerant microorganisms through rhizosphere activities. Therefore, a new halophyte/non-halophyte intercropping system is proposed on the basis of the rhizosphere response of two species to adversity. Liang et al. (2021) found that it significantly displayed higher crop productivity and salt removal in the cotton/halophyte intercropping [19]. However, Wang et al. showed that maize responded negatively in the suaeda/maize intercropping suaeda/maize, which might have been due to the N competition [18]. Whether or not the intercropping disadvantage of maize is caused by N competition should be further investigated.

Nitrogen is an important regulatory factor that acts on halophytes at different developmental stages in their life history [20,21]. Studies have shown that the NO_3_^−^-N application can promote the germination and vigor index of suaeda seeds [20,22]. Nitrogen application can significantly improve the nutrition status and the amount of osmotic regulatory substances, such as proline, soluble sugar, and Na^+^, to enhance salt tolerance [22]. Therefore, nitrogen might generate an important influence in the suaeda/maize intercropping. Nitrogen can obviously promote the accumulation of base ions in *S. salsa*, which effectively contributes to the remediation of saline soil in agriculture [21]. Due to the ability in accumulating salt of halophyte root, this migration process might be accelerated in the root zone of halophytes with nitrogen supply. Moreover, the differences in nitrogen form can affect soil ion exchange capacity, resulting in changes in soil chemical properties. Under salt stress, H^+^ may be secreted when halophytes absorb ammonium nitrogen (NH_4_^+^-N), which also has a competitive effect on the uptake of Ca^2+^, K^+^, and Mg^2+^. Moreover, OH^−^ is secreted, and Na^+^ may be aggravated for absorption due to nitrate (NO_3_^−^-N) application [23].

*S. salsa* (suaeda) is an annual euhalophyte with high aptitude in gathering Na^+^ to the root zone, being considered an ideal species for saline soil remediation in several studies [21,24]. *Z. mays* L. (maize) is a salt-sensitive non-halophyte with a fibrous root system that produces an important influence with a rhizosphere capacity in nutrient activation in the intercropping [25,26]. We speculate that the negative effect on maize in the suaeda/maize is caused by the interspecific competition for nitrogen. Therefore, we conducted the pot experiment and selected suaeda and maize as the research objects; following this, we set up three cropping patterns (suaeda/maize intercropping, suaeda monoculture, and maize monoculture) to detect the intercropping effect under the different nitrogen treatments. Here, we proposed the following hypotheses: (1) there is spatial complementarity and niche differentiation among roots to promote nutrition in this intercropping, and nitrogen supply may also attenuate this nutrient competition; and (2) in the suaeda/maize intercropping, nitrogen supply would promote the accumulation and uptake of salt ion in the roots of suaeda.

## 2. Results

### 2.1. Plant Biomass

**Figure 1:**
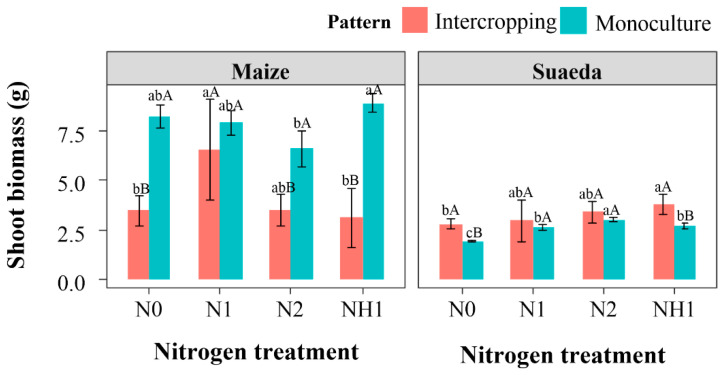
Effect of nitrogen treatment on aboveground biomass of *S. salsa* and *Z. mays* L. in the different cropping patterns. Capital letters indicate the significant differences between two cropping patterns. Lower case letters indicate significant differences of four N treatments.

The intercropping suaeda/maize significantly inhibited the aboveground growth of maize but promoted the aboveground growth of suaeda (Figure 1). Under N0 treatment, a dry weight unit of aboveground maize in the monoculture was 4.74 g higher than that in the intercropping. Moreover, in the intercropping, maize biomass under N1 treatment was significantly higher than that under N0 treatment, indicating that nitrogen alleviated the intercropping disadvantage of maize. While in the monoculture, the maize aboveground biomass was 1.00 g higher under NH1 treatment than that under N1 treatment. The contrary result was shown in the intercropping. It was found that nitrogen could significantly increase the aboveground biomass of suaeda in the monoculture (*p* < 0.05), but it displayed no significance in the intercropping.

### 2.2. Plant Ion Accumulation

**Figure 2:**
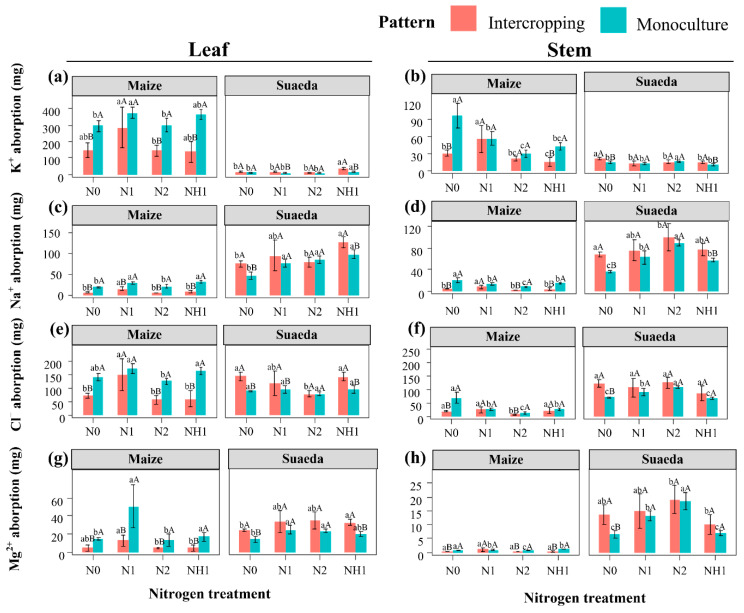
Effect of nitrogen treatment on K^+^ (**a**,**b**), Na^+^ (**c**,**d**), Cl^−^ (**e**,**f**), and Mg^2+^ (**g**,**h**) accumulation in leaves and stems of *S. salsa* and *Z. mays* L. in the different cropping patterns. Capital letters indicate the significant differences between two cropping patterns. Lower case letters indicate significant differences of four N treatments.

Ion accumulation is an important index used to evaluate halophyte improvement on saline–alkali soil. Under N0 treatment, for suaeda, the Na^+^ and Cl^−^ contents of leaves in the intercropping were 29.33 and 54.70 mg, respectively, being higher than that in the monoculture (Figure 2c,e). For maize, the ion accumulation of leaves in the monoculture was higher than that in the intercropping. In the intercropping, N1 treatment significantly enhanced the absorption of salt ions (K^+^, Na^+^, Cl^−^, and Mg^2+^) in the leaves of maize (*p* < 0.05), which were 138.35, 8.93, 77.40, and 8.44 mg higher than that under N0 treatment, respectively (Figure 2a,c,e,g). In the monoculture, N treatment promoted Na^+^ uptake of leaves for suaeda, and it was 38.71 mg higher under N2 treatment than that under N0 treatment (Figure 2c). In addition, in the monoculture, the accumulation of Na^+^ in the leaves of suaeda under NH1 treatment was significantly higher than that under N1 treatment.

Under N0 treatment, the accumulation of Na^+^ and Mg^2+^ in the stem of suaeda in the intercropping was significantly higher than that in monoculture (Figure 2b,h, *p* < 0.05), but for maize, it was lower in the intercropping than that in the monoculture. In the intercropping, compared to N0, N1 treatment significantly enhanced the K^+^, Na^+^, and Cl^−^ uptake in the stems of maize (*p* < 0.05) and promoted the Na^+^ accumulation in the stems of suaeda (Figure 2b,d,h). In the monoculture, N treatment significantly reduced the accumulation of K^+^, Na^+^, and Cl^−^ in maize stems, but promoted the accumulation of Na^+^, Cl^−^, and Mg^2+^ in suaeda stems.

### 2.3. Plant Nitrogen Accumulation

**Figure 3:**
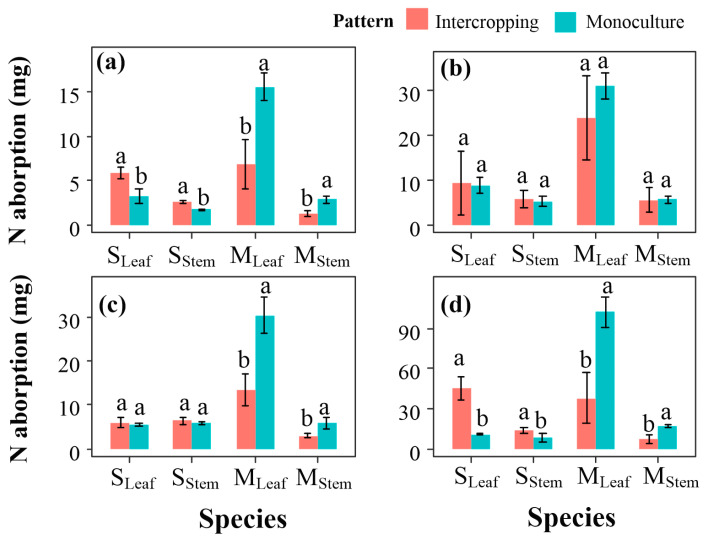
Effect of nitrogen treatment on N accumulation in leaves and stems of *S. salsa* and *Z. mays* L. in the cropping patterns. (**a**–**d**) N absorption under N0, N1, N2, and NH1 treatments, respectively. S_Leaf_ and S_team_ represent the N absorption of leaves and stems in suaeda, respectively, and M_Leaf_ and M_team_ represent the N absorption of leaves and stems in maize, respectively. Lower case letters indicate significant differences in N absorption between two cropping patterns.

Under N0 treatment, the nitrogen uptakes of the stems and leaves of suaeda in the intercropping were significantly, 0.95 and 2.63 mg, higher than those in the monoculture, while those of maize in the monoculture were 1.61 and 8.73 mg higher than those in the intercropping, respectively (Figure 3a). N1 treatment significantly increased nitrogen accumulation in intercropped maize (*p* < 0.05), while N2 and NH1 treatments had no obvious effect on nitrogen uptake of maize in the intercropping compared to that under N0 treatment (Figure 3b–d).

**Figure 4:**
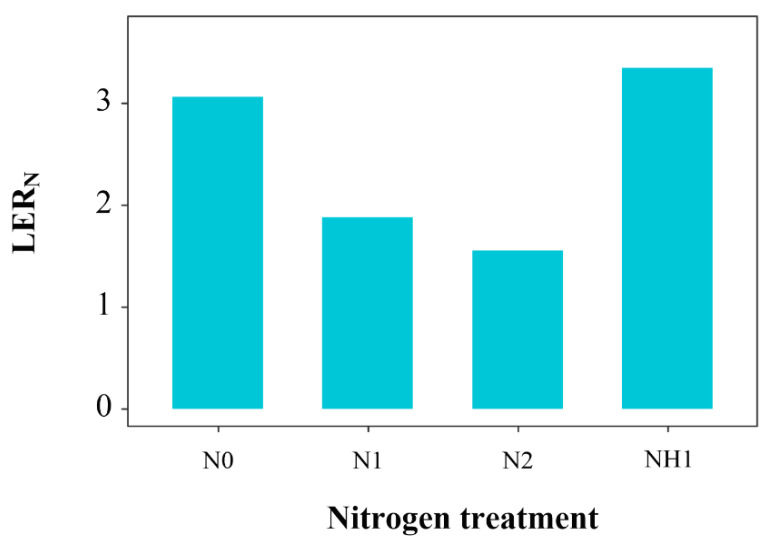
Variations in LERN values under the different N treatments.

LER_N_ is the relative competitive abilities of crops for nitrogen in the intercropping. The LER_N_ values under N0 and NH1 treatments were 3.07 and 3.35, respectively, which were higher than that under N1 and N2 treatments (Figure 4).

### 2.4. Soil Ion Content

**Figure 5:**
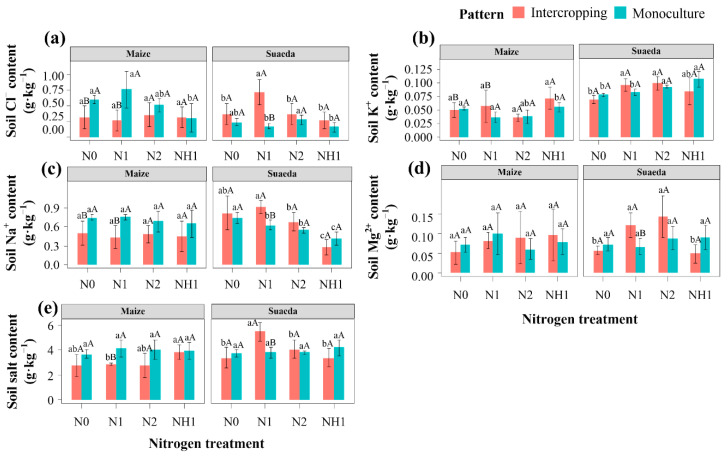
Effects of nitrogen on the content of Cl^−^ (**a**), K^+^ (**b**), Na^+^ (**c**), Mg2^+^ (**d**), and salt (**e**) in the rhizosphere soil of *S. salsa* and *Z. mays* L. in the different cropping patterns. Capital letters indicate the significant differences between two cropping patterns. Lower case letters indicate significant differences of four N treatments.

Under N0 treatment, the Na^+^ and Cl^−^ contents on maize side soil in the intercropping were 0.07 and 0.13 g·kg^−1^ lower than those in the monoculture, while they were 0.28 and 0.25 g·kg^−1^ higher on the suaeda sides in the intercropping than those in the monoculture, respectively (Figure 5a,c). The differences were more obvious with nitrogen treatment. Under N1 treatment, the contents of Na^+^ and Cl^−^ on the maize side in the intercropping were significantly lower than those in the monoculture (*p* < 0.05). In this intercropping, the Na^+^, Cl^−^, and salt contents of soil on the suaeda side were higher than those on the maize side (Figure 5). However, this difference in salt content between the sides of maize and suaeda was not obvious under NH1 treatment in the intercropping.

## 3. Discussion

In this study, it was found that the intercropping suaeda/maize would produce the disadvantage of maize growth, which was consistent with the results of Wang et al. (2022) [18]. This complex system was affected by uncertain factors, biotic as well as physical, that are essential for species development [27,28,29,30]. For instance, Zhang et al. (2019) found the complementarity between the yield disadvantage of vetch and the yield advantage of oats, due to a lack of complementarity for water acquisition [31]. The nutrient relationship between two plants in the intercropping tends to be a mutual–competitive relationship. Wang et al. (2022) found the soil nitrogen content to be reduced in the intercropping suaeda/maize, which suggested that the disadvantage of maize was caused by N competition [18]. In this study, N1 treatment could obviously promote the aboveground biomass of maize in the intercropping, not significantly different from that in the monoculture system (Figure 1), which also indicated that there was nutrient competition between maize and suaeda. Therefore, suitable nitrogen application obviously attenuated this nutrient competition in the suaeda and maize intercropping, which accurately corresponded to the first hypothesis. The negative effect on maize growth was bad for crop yield, which suggested that suitable cropping methods, such as inter-row distance and appropriate species to remove the disadvantage, should be figured out in subsequent studies, even in a wide range of salt conditions.

**Figure 6:**
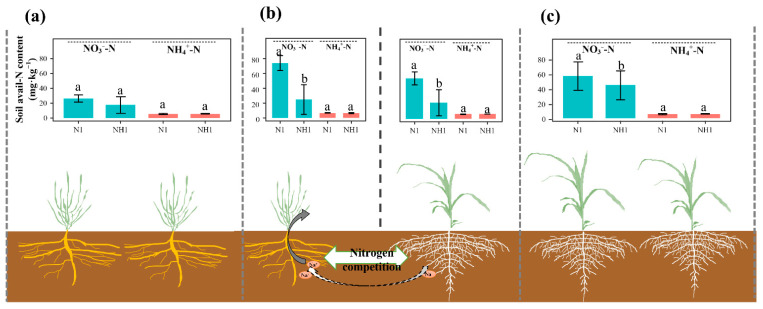
Variations in NO_3_^−^-N and NH_4_^+^-N content in the soil layer of *S. salsa* and *Z. mays* L. under N1 and NH1 treatments in the different cropping patterns. (**a**–**c**) Suaeda monoculture, suaeda/maize intercropping, and maize monoculture, respectively. Lower case letters indicate significant differences between two N treatments.

Previous studies found that maize has a strong preference for NH_4_^+^-N [32,33], which was also reflected in the monoculture (Figure 1). Hessini et al. (2019) found that NH_4_^+^-N favored maize growth by adjusting to salinity by accumulating inorganic solutes so as to improve the plant’s capacity osmotically, especially when exposed to saline conditions [34]. However, in this study, the effect of NH1 treatment on maize growth in the intercropping system was not obvious, being significantly lower than that of N1 treatment (Figure 1). This may have been due to the competition for NO_3_^−^-N with suaeda, which inhibited the growth of maize (Figure 6). It was explained that the LER_N_ value was 3.35 under NH1 treatment (Figure 4), and it was significantly higher than that under N0 treatment, which implied that the intercropping generated fierce competition for nitrogen between suaeda and maize under N0 and NH1 treatments.

*S. salsa* and *Z. mays* L. are two kinds of highly nitrogen-demanding plants, and insufficient nitrogen adversely affects their growth [20,21]. It was not to be ignored that, as the most direct exposure to adversity, root structure and function are terribly important to enhance the uptake in soil nutrients and accelerate microbial activities [35,36,37]. The root development and exudates are distinctly different, potentially producing important effects between the two species [38,39,40]. However, in the intercropping, the composition of root exudates and the presence of allelopathy still remain uncertain, needing to be further explored in further studies.

The aim of this study was to determine whether nitrogen contributes to salt migration from non-halophytes to halophytes in an intercropping system. The results showed that nitrogen treatment significantly promoted the accumulation of Na^+^ and salt around the root zone of suaeda (Figure 6c,e), which was consistent with the second hypothesis. This was mainly because the roots of suaeda have the capacity for absorbing and accumulating salt ion, and Na^+^ is a positive and critical factor for halotropism of the halophyte [9,10]. This also indicated that nitrogen application could promote the growth of maize in the intercropping system, possibly reducing the level of salt damage and improving the salt environment related to maize roots. However, the effects on plant phenotypes and related response mechanisms (e.g., relevant gene expression, antioxidant activity, and antioxidant enzymes) in deeply understanding the intercropping effect were not displayed in this study and should be explored in further studies.

## 4. Materials and Methods

### 4.1. Field Soil Properties

The soil was also obtained from the soil of tilled layer (0–20 cm) of Changji Hui Autonomous Prefecture (44°09′59′N, 87°04′56′E). Soil properties are as follows: pH 7.75, EC 1.32 dS·m^−1^, total salt content 0.50%, available nitrogen (NO_3_^−^-N and NH_4_^+^-N) 33.68 mg·kg^−1^, Olsen-P 4.62 mg·kg^−1^, and available potassium 0.25 g·kg^−1^. Here, in this study, the salt content was brought into correspondence with that of Wang et al. [18]. In addition, the optimal stress concentration of salt tolerance for maize was 0.5% by identification and evaluation [41]. The suaeda seeds were collected from the Karamay Halophyte Botanical Garden in October 2020, and the maize seeds were collected from Hejiayuan Agricultural Technology Co., LTD (Beijing, China).

### 4.2. Experimental Setup

**Figure 7:**
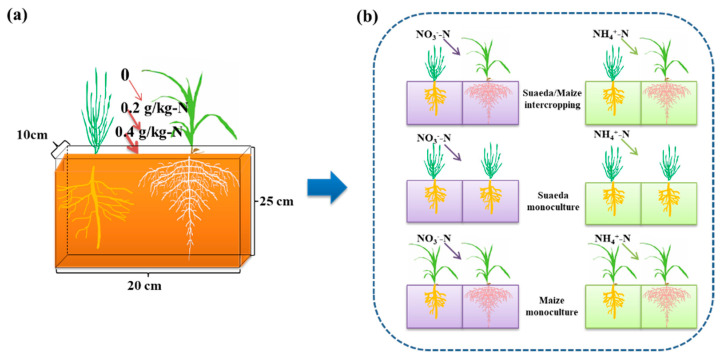
Experimental schematic diagram. (**a**) Experimental device. (**b**) Experimental procedure in the different cropping patterns.

The pot experiment was conducted in the greenhouse of Changji Huier Co., LTD (Changji, China), under a 14 h light/10 h dark photoperiod condition. This experimental system is a basin with a length of 20 cm, a width of 10 cm, and a height of 25 cm (Figure 7). Three cropping patterns were conducted as follows: suaeda/maize intercropping, maize monoculture, and suaeda monoculture. Moreover, five N treatments were set up as follows: N0, N1, N2, NH1, and NH2, defined as 0.0, 0.2, and 0.4 g·kg^−1^ NO_3_^−^-N, and 0.2 and 0.4 g·kg^−1^ NH_4_^+^-N, respectively. Each group had four replicates, with a total of 36 pots.

The 8 kg air-dried soil was mixed thoroughly with N fertilizer and then mixed with 3.36 g KH_2_PO_4_ to ensure the nutrient requirements of plant growth [42]. Each pot was filled with the mixed soil, and 1.6 L of distilled water was added into each pot for three days. Twenty seeds of suaeda were sown on one side of the pot and grew to 4 cm before thinning. After 15 days, four seeds of maize were sown on the other side of each pot. The weighing method was conducted to apply distilled water, which maintained the 20% field capacity (*w*/*w*) to dilute the influence of water on plant growth.

### 4.3. Sample Analysis

At approximately 45 days after sowing, the shoots of suaeda and maize were harvested in each pot and then were treated at 105 °C for 30 min and dried at 65 °C for 48 h to acquire the biomass. Shoot diluted extracts were measured for Na^+^, K^+^, Ca^2+^, and Mg^2+^ (Flame Photometer, 735 ICP-OES) and Cl^−^ (AgNO_3_ titration method). Shoot nitrogen (N) was extracted with 18 mol·L^−1^ H_2_SO_4_ and then measured by a Kieldahl Azotometer (Kjeltec 8420) and a UV spectrophotometer (CARY-60) [43].

The soil in the different patterns was sampled, dried, and passed through a 2 mm sieve. Soil available nitrogen was measured by the alkali hydrolysis diffusion method (5 g soil in 50 mL solution). Soil diluted extracts were analyzed for Na^+^, K^+^, Ca^2+^, Mg^2+^ (Flame Photometer, 735 ICP-OES), and Cl^−^ (AgNO_3_ titration method). In this study, we calculated the land equivalent ratio for nitrogen (LER_N_) [44,45]:

$${\mathrm{LER}}_{N}=\frac{NU_{\mathrm{IS}}}{NU_{\mathrm{MS}}}+\frac{NU_{\mathrm{IM}}}{NU_{\mathrm{MM}}}$$

where *NU*_IS_ represents the nitrogen uptake of suaeda in the intercropping, *NU*_MS_ represents the nitrogen uptake of suaeda in the monoculture, *NU*_IM_ represents the nitrogen uptake of maize in the intercropping, and *NU*_MM_ represents the nitrogen uptake of maize in the monoculture.

### 4.4. Data Analysis

We conducted an analysis of variance (ANOVA, one-way) to detect the influence on intercropping effects with nitrogen treatment using SPSS statistical software (SPSS version 19.0, IBM SPSS Inc., Chicago, IL, USA) and R software (version 4.0.3-win). Further, we investigated the differences in soil ion content between the layers on suaeda and maize sides among different nitrogen treatments. We detected the significant differences among means by the LSD test at the *p* < 0.05 probability level, which was manifested in different letters in the figures. Under the NH2 treatment, the germination of suaeda failed to survive, so the N treatment was divided into four treatments (N0, N1, N2, and NH1).

## 5. Conclusions

In this study, it was found that the suaeda/maize intercropping displayed a negative effect on maize that can be explained by interspecific ecological competition between suaeda and maize to a certain extent. Moderate nitrogen treatment produced a significant improvement on the biomass of maize, and salt ions gathered around the roots of suaeda. In the intercropping, the effect of NO_3_^−^-N treatment on the maize biomass was positive, while NH_4_^+^-N treatment displayed no significance. Moreover, the enhanced effect of NH_4_^+^-N on suaeda growth was displayed in the intercropping. This study could be greatly informative for determining the intercropping effect between suaeda and maize, providing a new insight with regard to improving saline land by phytoremediation.

## Data Availability

The data will be provided upon request by the corresponding author.

## Abbreviations

N: nitrogen; P, phosphorus; Olsen-P, available phosphorus; pH, pondus hydrogenii; EC, electrical conductivity.
